# Roles of DNA Damage Response Pathway in the Regulation of the Nuclear Envelope

**DOI:** 10.3390/cimb48030240

**Published:** 2026-02-24

**Authors:** Yasunao Kamikawa, Zuqian Wu, Kenshiro Fujise, Kazunori Imaizumi, Atsushi Saito

**Affiliations:** 1 Department of Biochemistry, Institute of Biomedical & Health Sciences, Hiroshima University, 1-2-3 Kasumi, Minami-ku, Hiroshima 734-8553, Hiroshima, Japan; 2 Department of Frontier Science and Interdisciplinary Research, Faculty of Medicine, Kanazawa University, 13-1 Takara-Machi, Kanazawa 920-8640, Ishikawa, Japan; 3 The Osaka Medical Research Foundation for Intractable Diseases, 3-7-11 Minamisumiyoshi, Sumiyoshi-ku, Osaka 558-0041, Osaka, Japan; 4 Department of Child Development and Molecular Brain Science, United Graduate School of Child Development, Osaka University, 2-2 Yamadaoka, Suita 565-0871, Osaka, Japan

**Keywords:** ATR, cell cycle, DNA damage response, lamin, nuclear envelope, nuclear envelope rupture, nuclear lamina

## Abstract

The nuclear envelope (NE) functions as a barrier between the cytoplasm and nucleus. Over the past decade, NE has revealed unexpectedly divergent structural alterations. NE rupture triggers the uncontrollable exchange of macromolecules across the NE and potentially causes DNA damage. Conversely, a recent study demonstrated that DNA damage induces NE rupture and that one of the major kinases in the DNA damage response (DDR) pathway, ataxia telangiectasia and Rad3-related protein, ATR, is a key molecule in these events. Here, we review the role of the DDR pathway in NE regulation, with a focus mainly on ATR.

## 1. Introduction

The nuclear envelope (NE) comprises two distinct bilayers, the inner (INM) and outer nuclear membranes (ONM), and numerous associated proteins [1]. There are three large protein complexes on the NE: the nuclear lamina, nuclear pore complex (NPC), and linker of the nucleoskeleton and cytoskeleton (LINC) complex [1]. The nuclear lamina, located underneath the INM, consists of two types of intermediate filament proteins: A-type (lamin A and lamin C) and B-type (lamin B1 and lamin B2) lamins [2]. The nuclear lamina provides structural support for the NE and is also involved in the regulation of the genome by direct and indirect interactions with some genomic regions, which often encode transcriptionally inactive genes and are referred to as lamina-associated domains [2,3]. NPCs include approximately 30 types of proteins, called nucleoporins (Nups), occupy the annular junctions between the INM and ONM, and function as gatekeepers for macromolecules that migrate between the cytoplasm and nucleus during interphase [1]. The LINC complex contains INM and ONM components, Sad1p/UNC-84 (SUN) domain proteins SUN1/2, and klarsicht/ANC-1/SYNE homology domain proteins Nesprins, respectively [1]. The LINC complex connects the cytoskeleton to NE by interacting with the nuclear lamina, thereby mediating mechanotransduction across the NE. In addition, several key proteins engage in the NE dynamics. The small protein barrier to autointegration factor (BAF) is associated with DNA and A-type lamins [4]. Lamina-associated polypeptide 1/2—Emerin—MAN1 (LEM)-domain proteins are transmembrane proteins on the INM and interact with BAF via their LEM-domain [4,5]. The endosomal sorting complex required for transport (ESCRT)-III, which mediates the topological remodeling of a variety of cellular membranes, plays a pivotal role in the reassembly of NE [6]. The NE-specific adaptor of ESCRT-III, Charged Multivesicular Body Protein 7 (CHMP7), is recruited to the NE by the LEM-domain proteins [6,7,8].

In this review, we introduce the roles of two major DDR kinase ATR and Ataxia-Telangiectasia Mutated (ATM) in the regulation of the NE and the response to NE rupture including the alterations in the NE, the nuclear actin filaments, and the chromatin remodeling. These processes are clinically relevant based on recent studies that demonstrate links between NE defects and a variety of disorders such as neurodegenerative diseases and cancers. evidences.

## 2. Structural Alteration of NE

The structure of NE undergoes drastic alterations during mitosis in every cell cycle. Here, we briefly introduce the molecular basis for the disassembly and reassembly of the NE before and after chromosome segregation in mammals. The disassembly of NE is triggered by the phosphorylation of NE-associated proteins, including BAF, lamins, and Nups, mainly mediated by Cyclin B/CDK1 and its downstream kinases [9]. This phosphorylation causes disassembly of the nuclear lamina and NPCs, and incorporation of the NE membrane into the endoplasmic reticulum together with INM proteins [9]. As a result, the membrane-containing components are dissociated from the chromosome, and Champion et al. showed that the removal of NE from the mitotic chromosome is essential for proper chromosome segregation [10]. Conversely, NE assembly is triggered by the dephosphorylation of these proteins via the inactivation of cyclin/CDK and/or activation of protein phosphatases. BAF is the earliest factor that accumulates on chromosomes at anaphase, leading to further recruitment of LEM-domain proteins and lamins [4,11]. In the late stage of NE assembly, ESCRT-III is targeted to the remaining holes in the NE by LEM-domain proteins and mediates NE closure [6,7,8,12].

In the last decade, non-canonical structural alterations in the NE outside of the cell cycle have been reported, including NE rupture and budding [13,14,15]. NE rupture has received considerable attention since Denais et al. and Raab et al. reported that NE is ruptured by cellular migration under confined conditions but is immediately repaired in a manner dependent on ESCRT-III [16,17]. One of the major consequences of NE rupture is DNA damage [16,17,18,19]. Mechanistically, mislocalization of the cytosolic DNase TREX-1 in the nucleus and/or reduced nuclear concentration of several key factors in DDR pathway have been proposed to cause NE rupture-dependent DNA damage [18,19]. Moreover, mouse models of Hutchinson-Gilford Progeria Syndrome (HGPS), a lethal progeroid syndrome caused by a mutation in the lamin A-encoding gene *LMNA*, and other disease-related mutations in NE-associated genes revealed that NE rupture leads to cellular dysfunction or loss of susceptible cells, such as vascular smooth muscle cells and skeletal muscle cells [20,21]. These findings demonstrated the pathological relevance of NE rupture in vivo.

**Figure 1:**
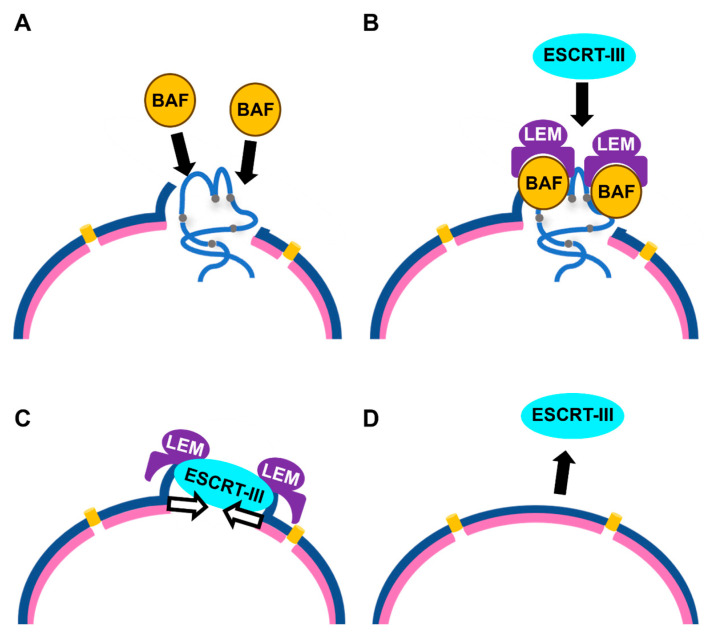
Molecular mechanism for NE repair. Upon NE rupture, BAF is accumulated to the ruptured site by binding to the DNA exposed to the cytoplasm (**A**). Consequently, LEM-domain proteins and ESCRT-III are recruited to the ruptured site (**B**,**C**) and ESCRT-III mediates NE closure (**C**). The dissolution of ESCRT-III is also important to maintain NE integrity (**D**). Blue half circle: NE, Pink half-circle: nuclear lamina. Yellow cylinder: NPC. The figure was illustrated by Microsoft PowerPoint for Microsoft 365 MSO.

NE rupture is usually repaired quickly, as evidenced by the recovery of compartmentalization between the cytoplasm and nucleus. A series of studies have revealed that NE repair shares a basic molecular mechanism with that of reassembly of the NE during mitosis [16,17,22,23,24]. Briefly, BAF accumulates at the ruptured sites by detecting leakage of genomic DNA into the cytoplasm; then, LEM-domain proteins and CHMP7 are sequentially recruited, leading to reseal of the NE by ESCRT-III (Figure 1). Subsequently, ESCRT-III is released from the repaired NE, which is also an important process to maintain the integrity of the NE (Figure 1) [16]. Meanwhile, BAF also counteracts Cyclic GMP-AMP Synthase (cGAS), which is a sensor for cytosolic DNA, and activates inflammatory signal by competitively binding to DNA at the ruptured sites [25]. Based on these similarities, it has been suggested that the NE repair pathway is also regulated in a cell cycle-dependent manner. Instead, a deficiency in the well-known tumor suppressors p53 or Rb causes an increased frequency of NE rupture [26]. In addition, it has been proposed that the NE repair efficiency varies during the cell cycle [27]. Furthermore, recent studies have revealed several unexpected results, such as the induction of NE rupture by DNA damage and the crucial roles of the DDR pathway in the regulation of NE structure and response to NE rupture [28,29,30,31,32]. Interestingly, ataxia telangiectasia and Rad3-related protein (ATR), one of the key kinases in the DDR, plays divergent roles in these events.

## 3. The Roles of DDR Pathway in NE Rupture and Its Consequences

DDR is indispensable for the maintenance of genome stability by repairing damaged DNA, permanent or transient cell cycle arrest, and/or elimination of cells with severe DNA damage by programmed cell death [33]. Three members of the phospho-inositide-3-kinase-related kinases, ATR, ATM, and DNA-dependent Protein Kinase (DNA-PK), are crucial DDR kinases that mediate signal transcription by phosphorylating many target proteins [33]. Their target amino acid sequences, which consist of serine (Ser) or threonine (Thr), followed by glutamine (Gln), and certain substrates are shared, although they reveal clear differences in substrate specificity and activation mechanisms. ATM and DNA-PK are activated by DNA double-strand breaks (DSBs) [33]. In contrast, ATR is activated by single-stranded (ss) DNA breaks or exposure of ssDNA, which can be generated as an intermediate in repairing diverse types of DNA damages [33]. ATR is the major DDR kinase involved in the S-phase checkpoint and response to DNA replication stress, in which the progression of the replication fork is challenged [34]. Consistently, ATR is essential in proliferating cells, whereas neither ATM nor DNA-PK is [33,35]. The roles of these kinases are mediated not only by direct phosphorylation but also by their targets such as Check Point Kinase (CHK) 1 and CHK2, which are phosphorylated by ATR and ATM, respectively [33]. In this section, we introduce the roles of ATR and ATM in the regulation of NE and the underlying molecular mechanisms. DNA-PK is not included because there is no report that demonstrate its direct involvement.

### 3.1. ATR-Dependent Alterations in NE

**Figure 2:**
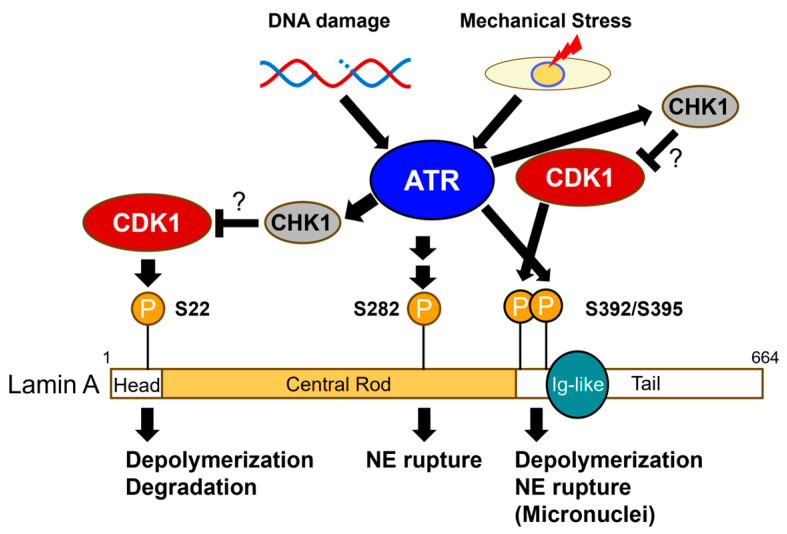
Regulation of the NE through phosphorylation of lamin A by ATR and CDK1. ATR is activated by mechanical stress and DNA damage. Lamin A is phosphorylated at several amino acid residues, including Ser22 and Ser395 by CDK1, leading to the depolymerization of lamin A filaments. Ser22 phosphorylation also causes its degradation. ATR directly phosphorylates Ser392 and promotes further phosphorylation of Ser395 by CDK1. Phosphorylation of Ser282 also requires ATR activity; however, the responsible kinase is unknown. ATR activates CHK1, which suppresses the kinase activity of CDK1. Numbers indicated: amino acid residues. The figure was illustrated by Microsoft PowerPoint for Microsoft 365 MSO.

Mechanical stimuli to the cells have a great impact on their fate and even trigger severe pathological conditions, such as cerebral hemorrhage and cardiac hypertrophy [36,37]. Given that the nucleus is the largest and the stiffest organelle, it can be readily imagined that the NE is affected by such mechanical stresses. Under normal conditions, ATR is localized throughout the nucleus [38]. Upon mechanical stimuli, such as hyperosmotic stress and mechanical compression, it shows mild accumulation in the NE [38]. The mechanisms underlying this shift in ATR localization have not yet been elucidated. Because a reduction in nucleoplasmic ATR and the dissociation of several nuclear proteins from chromatin have been observed under hyperosmotic conditions, its attenuated binding to the chromosome under stress conditions could be an explanation [38,39]. The major components of the nuclear lamina, lamins, consist of a globular N-terminal head domain, a central rod domain containing coiled coils, and a C-terminal tail domain harboring an immunoglobulin (Ig)-like domain (Figure 2) [2]. Lamins form a homodimer in a head-to-tail manner through parallel interactions between coiled coils and are further assembled into higher-order structures, which are thought to be disrupted by their phosphorylation during mitosis [2].

A member of the lamin family, lamin A is highly phosphorylated before disassembly of the NE during mitosis, including at Ser22 and Ser392 in humans (hereafter, all the indicated amino acid residues correspond to human proteins), which is mediated by CDK1, leading to the disruption of the filamentous structure of the nuclear lamina (Figure 2) [40,41]. Lamin A phosphorylation is also observed upon mechanical stimuli and DNA damage in interphase. Buxboim et al. demonstrated that the phosphorylation of lamin A at Ser22 negatively correlates with the stiffness of cell culture matrix and reduces nuclear stiffness by promoting the degradation of lamin A [42]. A series of recent studies have reported that ATR participates in lamin A-mediated regulation of NE in both direct and indirect manners [28,29]. Phosphorylation of lamin A on Ser392 is directly mediated by ATR in response to DNA replication stress, correlating with NE rupture in a subset of micronuclei, but not in primary nuclei (Figure 2) [29]. This phosphorylation is a prerequisite for further phosphorylation of lamin A at Ser395 by CDK1, which is deterministic for the destabilization of the NE of micronuclei, but does not affect the integrity of primary nuclei in this context (Figure 2). The differential sensitivity of primary nuclei and micronuclei to Ser392/Ser395 phosphorylation is likely due to the pre-existing structural defects of the NE in micronuclei. A substantial population of micronuclei revealed the loss or reduction of another member of lamin family lamin B1, suggesting the disorganization of the nuclear lamina [43]. Consistently, only lamin B1-negative micronuclei were likely subjected to ATR-dependent NE rupture. The expression level of lamin B1 is declined in cellular senescence induced by various stresses, such as oncogene expression or reactive oxygen species in vitro [44,45]. In addition, reduced expression of lamin B1 in vivo has been reported in mouse hippocampal neural stem cells associated with aging [46,47]. Given that the level of Ser392 phosphorylation is comparable between the primary nuclei and micronuclei, this phosphorylation may cause NE rupture under such conditions. In line with the sequential phosphorylation of lamin A, the mutant form of lamin A that causes HGPS, referred to as Progerin, harbors additional putative phosphorylation sites for ATR and CDK1 at Ser652 and Thr655, respectively, with a configuration similar to that of Ser392/Ser395 [48]. It has been reported that Progerin is phosphorylated at Ser628 and Ser636, where it is removed by proteolytic cleavage and thus not included in the mature form of wild type lamin A, by CK II and GSK-3b, and that inhibition of CK II causes extended life span in a HGPS model mice [49,50]. Thus, it would be interesting to investigate whether ATR-CDK1 phosphorylates Progerin at its C-terminus and contributes to HGPS pathology. Another question regarding this regulatory pathway is the negative regulation of CDK1 by ATR. Repressive phosphorylation of CDK1 at Thr14 and Tyrosine 15 should be removed for its full activation. A downstream kinase of ATR [51], CHK1 inhibits dephosphorylation of CDK1, enabling ATR to suppress CDK1 activity (Figure 2). Instead, Roy et al. recently reported that the mechanical stress-induced activation of ATR suppresses the phosphorylation of lamin A Ser22, leading to increased lamin A protein levels in mouse embryonic stem cells [52]. Such regulation may be important for coordinating the phosphorylation of lamin A and other NE-associated factors with the structural integrity and function of NE.

As mentioned above, DNA damage is a major consequence of NE rupture. In addition, many recent studies support the fact that DNA damage causes NE rupture. This is especially striking in p53-deficient cells and at least partially ATR-dependent [26,28,30]. Kovacs et al. showed that NE rupture is induced by DNA damage and coincides with the phosphorylation of several NE-associated proteins, including lamin A at Ser282 in an ATR-dependent manner (Figure 2) [28]. The amino acid sequence of this site did not match the consensus sequence for ATR, suggesting that a downstream kinase was responsible for this phosphorylation. CHK1 is likely not relevant to this event because its inhibition does not abolish NE rupture induced by DNA damage in this context. Kovacs et al. proposed that phosphorylation of lamin A at Ser282 leads to NE rupture, based on their observation that a substitution of Ser282 of lamin A to Ala suppresses DNA damage-induced NE rupture. They also showed that this mutation of lamin A at Ser282 compromised its interaction with Nesprin-2, suggesting that phosphorylation at Ser282 regulates mechanotransduction across the NE. However, because this mutation alone results in the aberrant aggregation of lamin A at the NE, its phenotypes should be carefully interpreted. Identification of the responsible kinase may be necessary to address this problem.

Another possible role of ATR in NE rupture is cell cycle arrest upon DNA damage. Ye et al. reported that DNA damage in M-phase due to DNA replication stress can cause NE rupture during the next interphase [30]. This is consistent with the fact that ATR is a major checkpoint regulator [51]; therefore, its inhibition causes failure of cell cycle arrest, and that p53-deficiency increases the frequency of NE rupture induced by DNA damage [28]. Mild DNA replication stress slows down the progression of the S-phase and per-turbs Cyclin/CDK, but does not complete cell cycle arrest, presumably resulting in dysregulation of cell cycle-dependent events including NE disassembly and reassembly. In such situations, NE may exhibit structural weaknesses after mitosis, which could cause NE rupture in daughter cells. This idea is also supported by the fact that severe nuclear deformation is observed upon depletion of ATR using siRNA for days, but not by short-term chemical inhibition of kinase activity [53]. Additional analyses using standardized methods and materials, including cell lines and DNA-damaging reagents, will greatly help understand the role of ATR in NE rupture and its response.

### 3.2. ATR and Nuclear Actin Filaments

**Figure 3:**
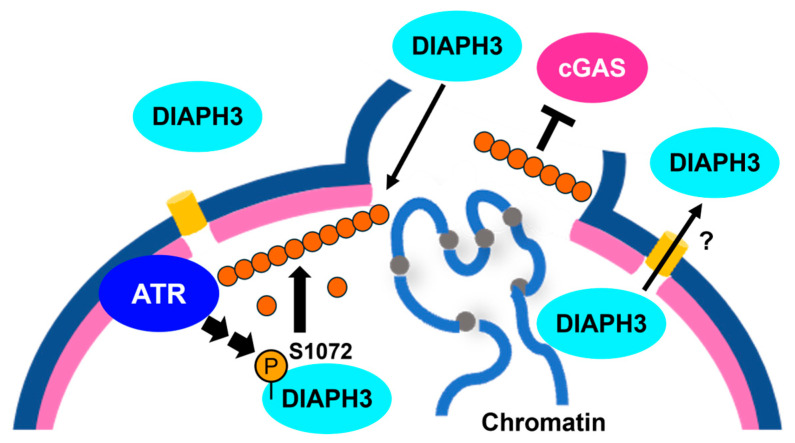
NE rupture causes nuclear localization of DIAPH3 and formation of nuclear actin filaments in a manner dependent on ATR activity. Under normal conditions, DIAPH3 is mainly localized to the cytoplasm, but is localized to the nucleus upon NE rupture. DIAPH3 is phosphorylated at Ser1072 and participates in nuclear actin filament formation. This phosphorylation requires ATR activity but may be mediated by different kinase(s). Nuclear actin filaments suppress chromatin leakage into cytoplasm. Once NE is repaired, DIAPH3 is exported from the nucleus. Blue half circle: NE. Pink half circle: nuclear lamina. Yellow cylinder: NPC. Isolated circles: monomeric actin. Connected circles: actin filaments. The figure was illustrated by Microsoft PowerPoint for Microsoft 365 MSO.

Actin forms filamentous structures in the nucleus, which play significant roles in a variety of molecular processes such as DNA replication, DNA repair, and transcription; however, its regulatory mechanisms are not yet fully understood [54]. Several distinct filamentous structures have been proposed in the nucleus based on the detectability of different types of probes [55]. Actin is imported into the nucleus via NPCs by the nuclear import factor Importin-9 in a complex with Cofilin, whereas its nuclear export is mediated by Exportin-6 in a complex with Profilin [56]. Nuclear transport of actin plays a pivotal role in the regulation of nuclear actin filaments, which can be perturbed by NE rupture. In addition to actin, some actin-regulating factors are also localized in the nucleus. Kamaras et al. demonstrated an increase in the nuclear fraction of a member of the Formin family, Diaphanous Related Formin (DIAPH) 3, in response to NE rupture, using time-lapse imaging of GFP-DIAPH3 [32]. In the microchannel, where cells are forced to migrate through a confined environment, GFP-DIAPH3 was evenly distributed in the cytoplasm and nucleus when the NE was ruptured, in contrast to its cytoplasmic localization under normal conditions (Figure 3). Interestingly, the localization pattern of GFP-DIAPH3 was immediately recovered once the nucleus passed the confined area, suggesting that DIAPH3 was actively exported from the nucleus to attenuate the formation of nuclear actin filaments (Figure 3). ATR activity is required for the formation of nuclear actin filaments upon NE rupture and the phosphorylation of DIAPH3 at Ser1072, although the kinase responsible for this was not identified (Figure 3). Depletion of DIAPH3, chemical inhibition of ATR, or expression of nuclear localization signal-fused actin non-polymerizable mutant R62D all resulted in increased chromatin leakage upon NE rupture, as evidenced by the increased cGAS signal at the leading edge of the nucleus in migrating cells. Collectively, these results indicate that ATR-DIAPH3 mediates the formation of actin filaments and suppresses chromatin leakage.

**Figure 4:**
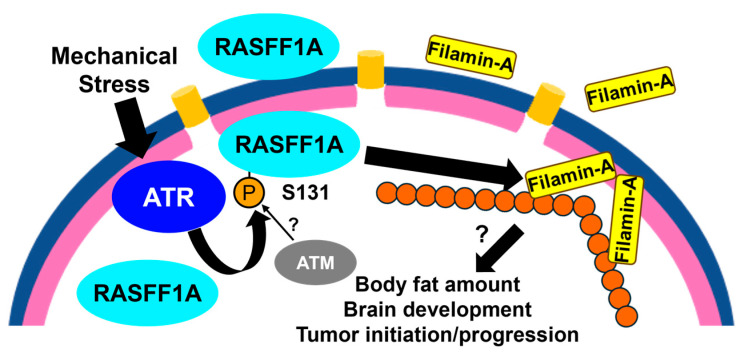
Phosphorylation of RASSF1A at Ser131 by ATR targets Filamin-A to the INM and promotes formation of nuclear actin filaments. RASSF1A is phosphorylated by ATR in response to mechanical stress. Consequently, an actin-binding protein, Filamin-A, is recruited to the INM, leading to the formation of nuclear actin filaments. Blue half circle: NE. Pink half circle: nuclear lamina. Connected circles: actin filaments. The figure was illustrated by Microsoft PowerPoint for Microsoft 365 MSO.

The formation of nuclear actin filaments is also induced by severe hyperosmotic stress and uniaxial stretching of cultured cells [31]. In this context, ATR contributes to the formation of actin filaments in the nucleus but phosphorylates different substrates such as RASSF1A at Ser131 [31]. RASSF1A is a well-known cancer-related gene whose promoter is frequently hypermethylated in a broad range of human cancers. Several lines of evidence support that RASSF1A functions as a tumor suppressor mediated by its divergent roles, including inhibition of cell cycle progression and suppression of stemness-related gene expression [57,58,59,60]. Phosphorylated RASSF1A revealed mild accumulation to the NE, whereas immunofluorescent signal against total RASSF1A was observed at entire nucleus and perinuclear regions (Figure 4) [31]. RASSF1A plays at least two distinct roles in regulating nuclear actin dynamics. First, RASSF1A is required to maintain the amount of nuclear actin, presumably via its interaction with Exportin-6 [60]. Depletion of RASSF1A leads to an increase in nuclear actin monomers. Second, phosphorylation of RASSF1A on Ser131 triggers the recruitment of Filamin-A, which crosslinks actin bundles in the cytoplasm, to the INM (Figure 4) [31,61]. Depletion of Filamin-A restrains the mechanical stress-induced formation of nuclear actin filaments, suggesting that INM localized Filamin-A is essential for this event. Filamin-A is cleaved by calpain in response to several stimuli, and the resulting C-terminal region is localized in the nucleus [62]. This form of nuclear Filamin-A participates in gene expression regulated by the androgen receptor [62]. Whether this C-terminal fragment or full-length Filamin-A is involved in the formation of nuclear actin filaments remains to be addressed. We speculate the latter case because the actin-binding domain is located in the N-terminal region of Filamin-A. Interestingly, a single amino acid substitution of RASSF1A at Ala133 to Ser, a human polymorphism of RASSF1A associated with susceptibility to several types of cancer including breast cancer and hepatocellular carcinoma, and poor survival in soft tissue sarcoma, results in reduced phosphorylation of RASSF1A at Ser131 [31,63,64]. Additionally, knock-in mice carrying this substitution showed increased body fat and defects in brain development, suggesting that RASSF1A phosphorylation at Ser131 contributes to its tumor suppressing activity, adipogenesis, and normal brain development [31]. Further investigations using knock-in mice carrying substitutions directly at Ser131 will answer this question more clearly. In addition, it should be noted that the regulation of the nuclear actin filaments contains divergent processes; both lamin A and lamin B can interact with actin filaments and expression of Progerin suppresses the formation of nuclear actin filaments, suggesting that the alterations in lamin A have substantial impact on nuclear actin filaments [65,66,67].

### 3.3. Roles of ATM in NE Regulation

ATM is also involved in the regulation of NE structure and dynamics. Notably, ATM deficiency under normal conditions results in the downregulation of lamin A at both mRNA and protein levels [68]. Although the expression pattern of lamin A is cell-type and tissue specific, its transcriptional regulation has not yet been elucidated. Instead, 3′-UTR of lamin A is targeted by a brain-specific microRNA (miRNA) miR-9, leading to the suppression of its expression in the brain especially in neural lineages, consistent with their low-level expression of lamin A [69]. While ATM participates in biogenesis of certain miRNAs in response to DNA damage, whether miR-9 is included in this criterion is not clear [70]. Either way, both ATM and ATR mediate the regulation of lamin A and presumably other NE-associated factors.

**Figure 5:**
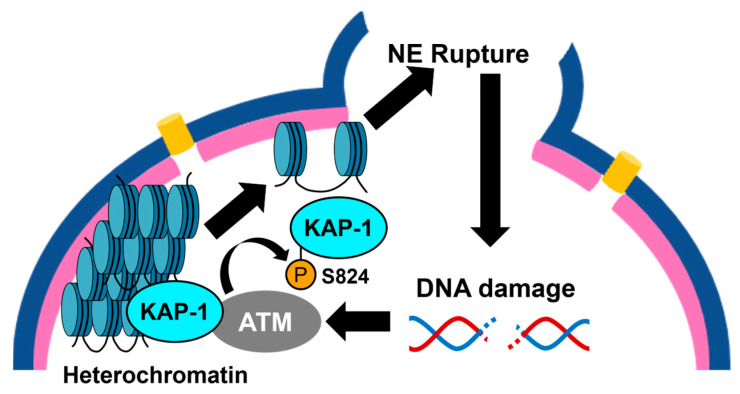
Regulation of NE and chromatin by ATM through phosphorylation of KAP-1. ATM is activated by DSB and phosphorylates KAP-1 at Ser824. Phosphorylated KAP-1 is dissociated from heterochromatin, leading to transition from closed chromatin to opened chromatin. Consequently, the nucleus is softened due to the reduced chromatin rigidity, leading to the NE rupture, which might trigger further DNA damage the activation of ATM, then loss of heterochromatin. Blue half circle: NE. Pink half circle: Nuclear lamina. The figure was illustrated by Microsoft PowerPoint for Microsoft 365 MSO.

Additionally, ATM modulates NE integrity by regulating chromatin status. Using microcapillary force measurement, Eskndir et al. demonstrated that DNA damage softens the nucleus but not the nuclear lamina in an ATM kinase activity dependent manner [71]. Although the underlaying mechanism for this regulation is not clear, previous studies revealed the significant roles of ATM in chromatin remodeling. ATM triggers heterochromatin loss in response to DNA damage by phosphorylating the factors involved in chromatin remodeling such as KAP-1, further recruiting regulators of heterochromatin including HP1 and SETDB1 [72,73,74,75]. ATM-dependent phosphorylation of KAP-1 at Ser824 reduces its binding capacity to heterochromatin, leading to transition of chromatin status from closed to opened (Figure 5) [74]. Based on the fact that NE rupture causes DNA damage, Eskndir et al. proposed positive feedback loop, in which elevated DNA damage by NE rupture further activates ATM and causes more relaxation of the chromatin (Figure 5). Conversely, it is possible that the negative feedback attenuates this reaction. For example, the formation of nuclear actin filaments in response to NE rupture can suppress nuclear softening in an ATR-dependent manner. In terms of the cross-talk between ATM and ATR, another remaining question is substrate specificities of these kinases. As described above, ATM and ATR share a subset of substrate. Indeed, ATM is responsible for the phosphorylation of RASSF1A at Ser131 in response to DNA damage but not for that of mechanical stress (Figure 4) [31]. To date, the roles of ATM in the regulation of nuclear actin filaments has not yet been elucidated. Whether ATM, ATR, and perhaps DNA-PK act separately or somehow cross-talk to regulate the NE should be addressed in the future.

## 4. Discussion

It is widely accepted that NE frequently ruptures and causes DNA damage, and evidence is still accumulating in many different contexts. Although it is reasonable to assume that NE rupture is frequently triggered by exogenous mechanical stress, there must be additional cues, especially in non-migrating cells in soft tissues such as mature neurons. As described above, DNA damage is one such type of stress and the DDR pathway play a substantial role in the regulation of NE integrity. In particular, the roles of ATR have been extensively investigated, presumably due to its attractive features: accumulation in the NE with mechanical stress. However, whether ATR actively accumulates to the NE has not yet been clarified and should be addressed in future studies. We speculate that the analysis using deletion mutants to identify the responsible region for its accumulation to the NE is a feasible approach, although handling of ATR, which consists of 2622 amino acid residues, could be problematic. It is also important to address the roles of ATR in NE; otherwise, it is quite challenging to separate its roles between DDR- and NE-related events.

Recently, several small molecule inhibitors of ATR have been investigated as potential anti-tumor treatments, with the aim of synthetic lethality together with other treatments, such as radiation and DNA-damaging reagents. As such, ATR inhibition can overcome resistance to the DNA crosslinking drug oxaliplatin in colorectal cancer cells [76]. This combined treatment increased cytosolic DNA and activated signals related to anti-tumor immunity, and in vivo analysis using a mouse model showed longer survival with the combined treatment. Together with the fact that ATR enhances the elimination of micronuclei with NE rupture and suppresses chromatin leakage in the primary nuclei, ATR inhibition may increase cytosolic DNA by suppressing these mechanisms. Therefore, a comprehensive understanding of these processes will enable us to control NE rupture and its consequences by modulating ATR, and contribute to the establishment of novel anti-tumor treatments that target NE.

## 5. Conclusions

The NE is the intersection of genomic DNA, proteins, and lipid membranes, therefore function as the molecular platform to integrate and transmit the divergent signals. Consistently, previous studies have illustrated links among specific factors such as those involved in DDR, NE dynamics, and the structures inside the nucleus (e.g., nuclear actin filament) as described in this review. We believe that further investigations with both higher resolution (single molecule observations and single cell analyses) and wider field of view (long-term time-lapse imaging, omics analyses, and in vivo observations) will elucidate the overall coordination among the NE, genomic DNA, and other organelles. The DDR pathway is one of the crucial targets to answer this question.

## Data Availability

No new data were created or analyzed in this study.

## Abbreviations

ATM	Ataxia Telangiectasia Mutated
ATR	Ataxia Telangiectasia and Rad3-related Protein
BAF	Barrier To Autointegration Factor
CDK	Cyclin Dependent Kinase
cGAS	Cyclic GMP-AMP Synthase
CHK	Checkpoint Kinase
CHMP	Charged Multivesicular Body Protein
CK	Casein Kinase
DIAPH	Diaphanous Related Formin
DDR	DNA Damage Response
ESCRT	Endosomal Sorting Complex Required for Transport
GSK	Glycogen Synthase Kinase
TREX	Three Prime Repair Exonuclease
HGPS	Hutchinson-Gilford Progeria Syndrome
HP	Heterochromatin Protein
INM	Inner Nuclear Membrane
KAP	KRAB-associated protein
LEM	Lamina-associated polypeptide 1/2―Emerin―MAN1
LINC	Linker of Nucleoskeleton and Cytoskeleton
NE	Nuclear Envelope
ONM	Outer Nuclear Membrane
RASSF1A	Ras Association Domain Family Member
SETDB	SET Domain Bifurcated Histone Lysine Methyltransferase

## References

[1] Hetzer M.W. (2010). The nuclear envelope. Cold Spring Harb. Perspect. Biol..

[2] Dechat T., Adam S.A., Taimen P., Shimi T., Goldman R.D. (2010). Nuclear Lamins. Cold Spring Harb. Perspect. Biol..

[3] Briand N., Collas P. (2020). Lamina-associated domains: Peripheral matters and internal affairs. Genome Biol..

[4] Kono Y., Shimi T. (2024). Crosstalk between mitotic reassembly and repair of the nuclear envelope. Nucleus.

[5] Barton L.J., Soshnev A.A., Geyer P.K. (2015). Networking in the nucleus: A spotlight on LEM-domain proteins. Curr. Opin. Cell Biol..

[6] Olmos Y., Carlton J. (2016). The ESCRT machinery: New roles at new holes. Curr. Opin. Cell Biol..

[7] Olmos Y., Perdrix-Rosell A., Carlton J.G. (2016). Membrane Binding by CHMP7 Coordinates ESCRT-III-Dependent Nuclear Envelope Reformation. Curr. Biol..

[8] Gu M., LaJoie D., Chen O.S., von Appen A., Ladinsky M.S., Redd M.J., Nikolova L., Bjorkman P.J., Sundquist W.I., Ullman K.S. (2017). LEM2 recruits CHMP7 for ESCRT-mediated nuclear envelope closure in fission yeast and human cells. Proc. Natl. Acad. Sci. USA.

[9] Ungricht R., Kutay U. (2017). Mechanisms and functions of nuclear envelope remodeling. Nat. Rev. Mol. Cell Biol..

[10] Champion L., Pawar S., Luithle N., Ungricht R., Kutay U. (2019). Dissociation of membrane-chromatin contacts is required for proper chromosome segregation in mitosis. Mol. Biol. Cell.

[11] Haraguchi T., Kojidani T., Koujin T., Shimi T., Osakada H., Mori C., Yamamoto A., Hiraoka Y. (2008). Live cell imaging and electron microscopy reveal dynamic processes of BAF-directed nuclear envelope assembly. J. Cell Sci..

[12] von Appen A., LaJoie D., Johnson I.E., Trnka M.J., Pick S.M., Burlingame A.L., Ullman K.S., Frost A. (2020). LEM2 phase separation promotes ESCRT-mediated nuclear envelope reformation. Nature.

[13] Vargas J.D., Hatch E.M., Anderson D.J., Hetzer M.W. (2012). Transient nuclear envelope rupturing during interphase in human cancer cells. Nucleus.

[14] Kamikawa Y., Imaizumi K. (2022). Advances in understanding the mechanisms of repairing damaged nuclear envelope. J. Biochem..

[15] Panagaki D., Croft J.T., Keuenhof K., Larsson Berglund L., Andersson S., Kohler V., Büttner S., Tamás M.J., Nyström T., Neutze R. (2021). Nuclear envelope budding is a response to cellular stress. Proc. Natl. Acad. Sci. USA.

[16] Denais C.M., Gilbert R.M., Isermann P., McGregor A.L., te Lindert M., Weigelin B., Davidson P.M., Friedl P., Wolf K., Lammerding J. (2016). Nuclear envelope rupture and repair during cancer cell migration. Science.

[17] Raab M., Gentili M., de Belly H., Thiam H.R., Vargas P., Jimenez A.J., Lautenschlaeger F., Voituriez R., Lennon-Duménil A.M., Manel N. (2016). ESCRT III repairs nuclear envelope ruptures during cell migration to limit DNA damage and cell death. Science.

[18] Nader G.P.d.F., Agüera-Gonzalez S., Routet F., Gratia M., Maurin M., Cancila V., Cadart C., Palamidessi A., Ramos R.N., San Roman M. (2021). Compromised nuclear envelope integrity drives TREX1-dependent DNA damage and tumor cell invasion. Cell.

[19] Pfeifer C.R., Xia Y., Zhu K., Liu D., Irianto J., García V.M.M., Millán L.M.S., Niese B., Harding S., Deviri D. (2018). Constricted migration increases DNA damage and independently represses cell cycle. Mol. Biol. Cell.

[20] Kim P.H., Chen N.Y., Heizer P.J., Tu Y., Weston T.A., Fong J.L.-C., Gill N.K., Rowat A.C., Young S.G., Fong L.G. (2021). Nuclear membrane ruptures underlie the vascular pathology in a mouse model of Hutchinson-Gilford progeria syndrome. JCI Insight.

[21] Earle A.J., Kirby T.J., Fedorchak G.R., Isermann P., Patel J., Iruvanti S., Moore S.A., Bonne G., Wallrath L.L., Lammerding J. (2020). Mutant lamins cause nuclear envelope rupture and DNA damage in skeletal muscle cells. Nat. Mater..

[22] Halfmann C.T., Sears R.M., Katiyar A., Busselman B.W., Aman L.K., Zhang Q., O’Bryan C.S., Angelini T.E., Lele T.P., Roux K.J. (2019). Repair of nuclear ruptures requires barrier-to-autointegration factor. J. Cell Biol..

[23] Young A.M., Gunn A.L., Hatch E.M. (2020). BAF facilitates interphase nuclear membrane repair through recruitment of nuclear transmembrane proteins. Mol. Biol. Cell.

[24] Kono Y., Adam S.A., Sato Y., Reddy K.L., Zheng Y., Medalia O., Goldman R.D., Kimura H., Shimi T. (2022). Nucleoplasmic lamin C rapidly accumulates at sites of nuclear envelope rupture with BAF and cGAS. J. Cell Biol..

[25] Guey B., Wischnewski M., Decout A., Makasheva K., Kaynak M., Sakar M.S., Fierz B., Ablasser A. (2020). BAF restricts cGAS on nuclear DNA to prevent innate immune activation. Science.

[26] Yang Z., Maciejowski J., de Lange T. (2017). Nuclear Envelope Rupture Is Enhanced by Loss of p53 or Rb. Mol. Cancer Res..

[27] Kamikawa Y., Wu Z., Nakazawa N., Ito T., Saito A., Imaizumi K. (2023). Impact of cell cycle on repair of ruptured nuclear envelope and sensitivity to nuclear envelope stress in glioblastoma. Cell Death Discov..

[28] Kovacs M.T., Vallette M., Wiertsema P., Dingli F., Loew D., Nader G.P.d.F., Piel M., Ceccaldi R. (2023). DNA damage induces nuclear envelope rupture through ATR-mediated phosphorylation of lamin A/C. Mol. Cell.

[29] Joo Y.K., Black E.M., Trier I., Haakma W., Zou L., Kabeche L. (2023). ATR promotes clearance of damaged DNA and damaged cells by rupturing micronuclei. Mol. Cell.

[30] Ye G., He Y., Zhang Y., Li D., Liu F., Li Y., Ge Q., Guo Q., Han S., Song C. (2025). Mitotic DNA repair by TMEJ suppresses replication stress-induced nuclear envelope reassembly defect. Nat. Commun..

[31] Chatzifrangkeskou M., Stanly T., Koennig D., Campos-Soares L., Eyres M., Hasson A., Perdiou A., Vendrell I., Fischer R., Das S. (2025). ATR-hippo drives force signaling to nuclear F-actin and links mechanotransduction to neurological disorders. Sci. Adv..

[32] Kamaras C., Frank D., Wang H., Drepper F., Huesgen P.F., Grosse R. (2025). Nuclear rupture in confined cell migration triggers nuclear actin polymerization to limit chromatin leakage. EMBO J..

[33] Blackford A.N., Jackson S.P. (2017). ATM, ATR, and DNA-PK: The Trinity at the Heart of the DNA Damage Response. Mol. Cell.

[34] Saldivar J.C., Cortez D., Cimprich K.A. (2017). The essential kinase ATR: Ensuring faithful duplication of a challenging genome. Nat. Rev. Mol. Cell Biol..

[35] Brown E.J., Baltimore D. (2000). ATR disruption leads to chromosomal fragmentation and early embryonic lethality. Genes. Dev..

[36] Ariyada K., Yamagishi K., Kihara T., Muraki I., Imano H., Kokubo Y., Saito I., Yatsuya H., Iso H., Tsugane S. (2024). Risk factors for intracerebral hemorrhage by five specific bleeding sites: Japan Public Health Center-based Prospective Study. Eur. Stroke J..

[37] Kuwahara K., Ohkubo T., Inoue Y., Honda T., Yamamoto S., Nakagawa T., Okazaki H., Yamamoto M., Miyamoto T., Gommori N. (2024). Blood pressure classification using the Japanese Society of Hypertension Guidelines for the Management of Hypertension and cardiovascular events among young to middle-aged working adults. Hypertens. Res..

[38] Kumar A., Mazzanti M., Mistrik M., Kosar M., Beznoussenko G.V., Mironov A.A., Garrè M., Parazzoli D., Shivashankar G.V., Scita G. (2014). ATR mediates a checkpoint at the nuclear envelope in response to mechanical stress. Cell.

[39] Schoborg T., Rickels R., Barrios J., Labrador M. (2013). Chromatin insulator bodies are nuclear structures that form in response to osmotic stress and cell death. J. Cell Biol..

[40] Heald R., McKeon F. (1990). Mutations of phosphorylation sites in lamin A that prevent nuclear lamina disassembly in mitosis. Cell.

[41] Peter M., Heitlinger E., Häner M., Aebi U., Nigg E.A. (1991). Disassembly of in vitro formed lamin head-to-tail polymers by CDC2 kinase. EMBO J..

[42] Buxboim A., Swift J., Irianto J., Spinler K.R., Dingal P.C.D.P., Athirasala A., Kao Y.-R.C., Cho S., Harada T., Shin J.-W. (2014). Matrix elasticity regulates lamin-A,C phosphorylation and turnover with feedback to actomyosin. Curr. Biol..

[43] Hatch E.M., Fischer A.H., Deerinck T.J., Hetzer M.W. (2013). Catastrophic Nuclear Envelope Collapse in Cancer Cell Micronuclei. Cell.

[44] Shimi T., Butin-Israeli V., Adam S.A., Hamanaka R.B., Goldman A.E., Lucas C.A., Shumaker D.K., Kosak S.T., Chandel N.S., Goldman R.D. (2011). The role of nuclear lamin B1 in cell proliferation and senescence. Genes Dev..

[45] Freund A., Laberge R.-M., Demaria M., Campisi J. (2012). Lamin B1 loss is a senescence-associated biomarker. Mol. Biol. Cell.

[46] Bedrosian T.A., Houtman J., Eguiguren J.S., Ghassemzadeh S., Rund N., Novaresi N.M., Hu L., Parylak S.L., Denli A.M., Randolph-Moore L. (2021). Lamin B1 decline underlies age-related loss of adult hippocampal neurogenesis. EMBO J..

[47] Bin Imtiaz M.K., Jaeger B.N., Bottes S., Machado R.A.C., Vidmar M., Moore D.L., Jessberger S. (2021). Declining lamin B1 expression mediates age-dependent decreases of hippocampal stem cell activity. Cell Stem Cell.

[48] Davies B.S.J., Fong L.G., Yang S.H., Coffinier C., Young S.G. (2009). The posttranslational processing of prelamin A and disease. Annu. Rev. Genom. Hum. Genet..

[49] Ao Y., Wu Z., Liao Z., Lan J., Zhang J., Sun P., Liu B., Wang Z. (2023). Role of C-Terminal Phosphorylation of Lamin A in DNA Damage and Cellular Senescence. Cells.

[50] Ao Y., Zhang J., Liu Z., Qian M., Li Y., Wu Z., Sun P., Wu J., Bei W., Wen J. (2019). Lamin A buffers CK2 kinase activity to modulate aging in a progeria mouse model. Sci. Adv..

[51] Sørensen C.S., Syljuåsen R.G. (2012). Safeguarding genome integrity: The checkpoint kinases ATR, CHK1 and WEE1 restrain CDK activity during normal DNA replication. Nucleic Acids Res..

[52] Roy T., Ghosh S., Piplani N., Sthanam L.K., Tiwary N., Dhar S., Konyak W.C.W., Panigrahi S.S., Singh P., Sowpati D.T. (2025). Nuclear compression-mediated DNA damage drives ATR-dependent Lamin expression and mouse ESC differentiation. Nucleic Acids Res..

[53] Kidiyoor G.R., Li Q., Bastianello G., Bruhn C., Giovannetti I., Mohamood A., Beznoussenko G.V., Mironov A., Raab M., Piel M. (2020). ATR is essential for preservation of cell mechanics and nuclear integrity during interstitial migration. Nat. Commun..

[54] Ulferts S., Lopes M., Miyamoto K., Grosse R. (2024). Nuclear actin dynamics and functions at a glance. J. Cell Sci..

[55] Melak M., Plessner M., Grosse R. (2017). Actin visualization at a glance. J. Cell Sci..

[56] Fernandez M.K., Sinha M., Zidan M., Renz M. (2024). Nuclear actin filaments—A historical perspective. Nucleus.

[57] Grawenda A.M., O’Neill E. (2015). Clinical utility of RASSF1A methylation in human malignancies. Br. J. Cancer.

[58] Donninger H., Vos M.D., Clark G.J. (2007). The RASSF1A tumor suppressor. J. Cell Sci..

[59] Pankova D., Jiang Y., Chatzifrangkeskou M., Vendrell I., Buzzelli J., Ryan A., Brown C., O’Neill E. (2019). RASSF1A controls tissue stiffness and cancer stem-like cells in lung adenocarcinoma. EMBO J..

[60] Chatzifrangkeskou M., Pefani D., Eyres M., Vendrell I., Fischer R., Pankova D., O’Neill E. (2019). RASSF1A is required for the maintenance of nuclear actin levels. EMBO J..

[61] van der Flier A., Sonnenberg A. (2001). Structural and functional aspects of filamins. Biochim. Biophys. Acta.

[62] Loy C.J., Sim K.S., Yong E.L. (2003). Filamin-A fragment localizes to the nucleus to regulate androgen receptor and coactivator functions. Proc. Natl. Acad. Sci. USA.

[63] Gao B., Xie X.-J., Huang C., Shames D.S., Chen T.T.-L., Lewis C.M., Bian A., Zhang B., Olopade O.I., Garber J.E. (2008). RASSF1A polymorphism A133S is associated with early onset breast cancer in BRCA1/2 mutation carriers. Cancer Res..

[64] Yee K.S., Grochola L., Hamilton G., Grawenda A., Bond E.E., Taubert H., Wurl P., Bond G.L., O’Neill E. (2012). A RASSF1A Polymorphism Restricts p53/p73 Activation and Associates with Poor Survival and Accelerated Age of Onset of Soft Tissue Sarcoma. Cancer Res..

[65] Sasseville A.M.-J., Langelier Y. (1998). In vitro interaction of the carboxy-terminal domain of lamin A with actin. FEBS Lett..

[66] Simon D.N., Zastrow M.S., Wilson K.L. (2010). Direct actin binding to A- and B-type lamin tails and actin filament bundling by the lamin A tail. Nucleus.

[67] Takahashi Y., Hiratsuka S., Machida N., Takahashi D., Matsushita J., Hozak P., Misteli T., Miyamoto K., Harata M. (2020). Impairment of nuclear F-actin formation and its relevance to cellular phenotypes in Hutchinson-Gilford progeria syndrome. Nucleus.

[68] Shah P., McGuigan C.W., Cheng S., Vanpouille-Box C., Demaria S., Weiss R.S., Lammerding J. (2022). ATM Modulates Nuclear Mechanics by Regulating Lamin A Levels. Front. Cell Dev. Biol..

[69] Jung H.-J., Coffinier C., Choe Y., Beigneux A.P., Davies B.S.J., Yang S.H., Barnes R.H., Hong J., Sun T., Pleasure S.J. (2012). Regulation of prelamin A but not lamin C by miR-9, a brain-specific microRNA. Proc. Natl. Acad. Sci. USA.

[70] Zhang X., Wan G., Berger F.G., He X., Lu X. (2011). The ATM kinase induces microRNA biogenesis in the DNA damage response. Mol. Cell.

[71] Eskndir N., Hossain M., Currey M.L., Pho M., Berrada Y., Lin K., Manning G., Prince K., Stephens A.D. (2025). DNA damage causes ATM-dependent heterochromatin loss leading to nuclear softening, blebbing, and rupture. Mol. Biol. Cell.

[72] Ryan R.F., Schultz D.C., Ayyanathan K., Singh P.B., Friedman J.R., Fredericks W.J., Rauscher F.J. (1999). KAP-1 corepressor protein interacts and colocalizes with heterochromatic and euchromatic HP1 proteins: A potential role for Krüppel-associated box-zinc finger proteins in heterochromatin-mediated gene silencing. Mol. Cell Biol..

[73] Schultz D.C., Ayyanathan K., Negorev D., Maul G.G., Rauscher F.J. (2002). SETDB1: A novel KAP-1-associated histone H3, lysine 9-specific methyltransferase that contributes to HP1-mediated silencing of euchromatic genes by KRAB zinc-finger proteins. Genes Dev..

[74] Ziv Y., Bielopolski D., Galanty Y., Lukas C., Taya Y., Schultz D.C., Lukas J., Bekker-Jensen S., Bartek J., Shiloh Y. (2006). Chromatin relaxation in response to DNA double-strand breaks is modulated by a novel ATM- and KAP-1 dependent pathway. Nat. Cell Biol..

[75] Goodarzi A.A., Noon A.T., Deckbar D., Ziv Y., Shiloh Y., Löbrich M., Jeggo P.A. (2008). ATM signaling facilitates repair of DNA double-strand breaks associated with heterochromatin. Mol. Cell.

[76] Combès E., Andrade A.F., Tosi D., Michaud H.-A., Coquel F., Garambois V., Desigaud D., Jarlier M., Coquelle A., Pasero P. (2019). Inhibition of Ataxia-Telangiectasia Mutated and RAD3-Related (ATR) Overcomes Oxaliplatin Resistance and Promotes Antitumor Immunity in Colorectal Cancer. Cancer Res..

